# New Tools for Embryo Selection: Comprehensive Chromosome Screening by Array Comparative Genomic Hybridization

**DOI:** 10.1155/2014/517125

**Published:** 2014-04-29

**Authors:** Lorena Rodrigo, Emilia Mateu, Amparo Mercader, Ana Cristina Cobo, Vanessa Peinado, Miguel Milán, Nasser Al-Asmar, Inmaculada Campos-Galindo, Sandra García-Herrero, Pere Mir, Carlos Simón, Carmen Rubio

**Affiliations:** ^1^IVIOMICS SPAIN, Parc Cientific Universitat de València, Calle Catedrático Agustín Escardino 9, 46980 Valencia, Spain; ^2^Instituto Valenciano de Infertilidad (IVI), Instituto Universitario IVI/INCLIVA, Plaza de la Policía Local 3, 46015 Valencia, Spain; ^3^IVIOMICS USA, 1200 NW 78th Avenue, Suite 103, Miami, FL 33126, USA; ^4^IVIOMICS INDIA, 13 Olof Palme Marg, Vasant Vihar, New Delhi 11057, India; ^5^Fundación IVI, 46980 Valencia, Spain; ^6^Department of Obstetrics and Gynecology, Stanford University School of Medicine, Stanford, CA 94305, USA

## Abstract

The objective of this study was to evaluate the usefulness of comprehensive chromosome screening (CCS) using array comparative genomic hybridization (aCGH). The study included 1420 CCS cycles for recurrent miscarriage (*n* = 203); repetitive implantation failure (*n* = 188); severe male factor (*n* = 116); previous trisomic pregnancy (*n* = 33); and advanced maternal age (*n* = 880). CCS was performed in cycles with fresh oocytes and embryos (*n* = 774); mixed cycles with fresh and vitrified oocytes (*n* = 320); mixed cycles with fresh and vitrified day-2 embryos (*n* = 235); and mixed cycles with fresh and vitrified day-3 embryos (*n* = 91). Day-3 embryo biopsy was performed and analyzed by aCGH followed by day-5 embryo transfer. Consistent implantation (range: 40.5–54.2%) and pregnancy rates per transfer (range: 46.0–62.9%) were obtained for all the indications and independently of the origin of the oocytes or embryos. However, a lower delivery rate per cycle was achieved in women aged over 40 years (18.1%) due to the higher percentage of aneuploid embryos (85.3%) and lower number of cycles with at least one euploid embryo available per transfer (40.3%). We concluded that aneuploidy is one of the major factors which affect embryo implantation.

## 1. Introduction

Aneuploidies are common in early human embryos [1, 2]. Trisomic and monosomic embryos account for at least 10% of human pregnancies and, for women nearing the end of their reproductive lifespan, the incidence may exceed 50% [3]. Further, aneuploidy rates are higher in oocytes and embryos from women with advanced maternal age (AMA) [4] which probably stems from meiotic recombination defects exacerbated by age [5]. Recent studies in humans and model organisms have shed new light on the complexity of meiotic defects, providing evidence that the age-related increase in errors in human females is not attributable to a single factor but to an interplay between the unique features of oogenesis and a host of other endogenous and exogenous factors [3]. Age-related defects result in higher aneuploidy rates in offspring and an increase in spontaneous abortions, thereby reducing ongoing implantation rates [6]. Aneuploidy may also be a contributing factor in other infertile populations; for example, despite other potential causes, an abnormal embryonic karyotype was found to be the most frequent cause of recurrent miscarriage (RM) [7]. In the same study, the percentage of cases with RM of truly unexplained origin was limited to 24.5%. While the diagnosis of repetitive implantation failure (RIF) remains a clinical challenge (its causes can be multiple, often with ill-defined embryonic and endometrial contributing factors), embryonic aneuploidy has been proposed as one of the leading embryonic causes [8]. In male factor (MF) infertility, an increase in sperm chromosomal abnormalities due to impairment of the meiotic process has been described [9, 10]. Additionally, a higher incidence of abnormal karyotypes has been described in the miscarriages of couples undergoing intracytoplasmic sperm injection (ICSI) because of MF infertility [11].

Despite a meta-analysis compiling nine randomized controlled clinical trials (RCTs) [12] indicating that there is no benefit to preimplantation genetic screening (PGS) by fluorescence* in situ* hybridization (FISH) for a limited number of chromosomes, some controversial opinions have surfaced regarding the convenience for embryo aneuploidy screening [13–19], including our own experience which differs from previously published studies. We conducted two prospective, randomized trials to evaluate the usefulness of PGS in AMA patients between 41 and 44 years of age and RIF patients aged less than 40 years of age. We observed a significant increase in the live birth rates in the PGS group compared to the blastocyst group in the AMA study (32.3% versus 15.5%; *P* = 0.0099) and a clear trend towards increased live birth rates in the RIF study (47.9% versus 27.9%). We therefore concluded that PGS with classic FISH is beneficial for these two indications if proper blastomere biopsy procedures and good laboratory conditions are applied [20].

Despite our findings, there is still a clear need for a technique capable of comprehensive chromosome screening (CCS), which could also produce reliable and faithful results in a short period of time. The first approach was comparative genomic hybridization (CGH), and several studies were published using this technology [21–23]. However, in the last three years, embryo aneuploidy screening has evolved: it is more broadly applied in* in vitro* fertilization (IVF) programs and now includes other approaches that allow results to be obtained in a shorter period of time, such as oligoarrays, single nucleotide polymorphism arrays, quantitative PCR, and CGH bacterial artificial chromosome arrays (SNP, qPCR, and CGH BAC, resp.) [24–33]. In two recently published reviews, array-CGH (aCGH) was described as a robust and accessible diagnostic approach to assess 24-chromosome aneuploidy, and hence IVF programs are moving towards PGS using aCGH [34, 35].

Independent of the type of platform used, the technique selected for screening all 24 chromosomes should offer reliable and timely results and should only be applied in clinical programs after validation with an already well-established technique. In our program, we first validated the aCGH platform in single cells from embryos previously diagnosed as abnormal by FISH, obtaining similar error rates below three percent for both techniques [36]. Next, we confirmed that there were no differences in efficiency and accuracy when comparing day-3 and day-5 whole embryo array analysis [30]. This was further validated in another study using the same aCGH platform confirming the high efficiency of the platform: only 2.9% of embryos had no results, and the error rate when compared to FISH was 1.9% [37]. In the work presented here, we describe our current experience with the clinical application of CCS using aCGH for different clinical indications, considering oocyte and embryo vitrification as a coadjuvant technique to improve reproductive outcomes in IVF patients.

## 2. Materials and Methods

### 2.1. Patients

This retrospective study compiled 1420 cycles with a day-3 biopsy in which aCGH analysis was performed, from February 2010 to February 2013. Clinical indications for CCS were the following: RM (two or more miscarriages of unknown etiology); RIF (three or more previous IVF failures); MF (low sperm concentration or a significant increase in sperm chromosomal abnormalities); couples with a previous trisomic pregnancy (PTP); and AMA (40 years or older). The study included different cycle types: cycles in which all oocytes and embryos came from fresh cycles (*n* = 774); mixed cycles with fresh and vitrified oocytes (*n* = 320); mixed cycles with fresh and vitrified day-2 embryos (*n* = 235); and mixed cycles with fresh and vitrified day-3 embryos (*n* = 91). The goal of vitrification at different stages was to increase the number of embryos for the analysis within a single CCS cycle.

### 2.2. Embryo Biopsy and Culture Conditions

Patients underwent ovarian stimulation using standardized protocols. When at least two follicles reached 18 mm in diameter, recombinant human chorionic gonadotropin (hCG, Ovitrelle, 250 mg, Merck Serono, Geneva, Switzerland) was administered, and oocyte retrieval was scheduled 36 hours later. ICSI was performed in all cases [38]. Fertilization was assessed 17–20 hours after microinjection, and embryo cleavage was recorded every 24 hours. The CCS cycles were performed in different IVF centers using two main protocols. In most centers, embryos were grown in IVF/CCM medium (1/1) (Vitrolife, Göteborg, Sweden) until day 3 and were subsequently cultured in CCM medium with a monolayer of endometrial epithelial cells until day 5 [39]. In the remaining centers, global sequential culture system (LifeGlobal, Guilford, CT) was used with tri-gas incubators (7% O_2_ and 5% CO_2_).

Embryo biopsy was performed on day 3 and can be summarized as follows: embryos were placed on a droplet containing Ca^2+^/Mg^2+^-free medium (G-PGD, Vitrolife, Göteborg, Sweden/LifeGlobal, Guilford, CT), the* zona pellucida* was perforated using laser technology (OCTAX, Herborn, Germany), and one blastomere was withdrawn from each embryo. Only embryos with five or more nucleated blastomeres and less than 25% fragmentation were biopsied. Individual blastomeres were placed in 0.2 mL PCR tubes containing 2 *μ*L PBS. For blastomere washing and handling, 1% polyvinylpyrrolidone (PVP) was used. Properly developed euploid embryos were transferred on day 5, and surplus euploid embryos were vitrified either on day 5 or on day 6.

### 2.3. Oocyte and Embryo Vitrification

The Cryotop method was used as previously described by Kuwayama et al. (2005) [40] and adapted for our laboratory [41]. In brief, oocytes/embryos were immersed in a solution containing 7.5% (v/v) ethylene glycol (EG) with 7.5% (v/v) dimethylsulfoxide (DMSO) in TCM199 medium with 20% (v/v) synthetic serum substitute (SSS) at room temperature for 15 minutes. Subsequently, oocytes/embryos were placed in a solution containing 15% EG with 15% DMSO and 0.5 mol/L sucrose. One minute later, they were placed on the Cryotop strip and immediately submerged in filtered liquid nitrogen (Brymill filter model 9409, Brymill Corporation, Ellington, CT, USA). For warming, the Cryotop was removed from the liquid nitrogen and instantly placed in 1.0 M sucrose in TCM199 with 20% SSS at 37°C. After 1 minute, oocytes/embryos were placed in 0.5 mol/L sucrose in TCM199 with 20% SSS at room temperature for 3 minutes. Finally, two consecutive washes (5 minutes and 1 minute) were performed with TCM199 with 20% SSS at room temperature before oocytes were incubated at 37°C for two hours preceding ICSI.

### 2.4. DNA Amplification and Array-Comparative Genomic Hybridization Protocol

To perform day-3 aCGH analysis, a single cell from each embryo was amplified using the Sureplex DNA amplification system (BlueGnome, Cambridge, UK). Amplification quality was ensured by gel electrophoresis (Lonza, Rockland, USA). Sample and control DNA were labelled with Cy3 and Cy5 fluorophores following the manufacturer's instructions. Labelling mixes were combined and hybridized on 24sure arrays (V2 and V3, BlueGnome, Cambridge, UK) for 6–12 hours. Each probe is specific to a different chromosomal region and occupies a discrete spot on the slide. Chromosomal loss or gain is revealed by the color adopted by each spot after hybridization. The technique involves the competitive hybridization of differentially labeled test and reference DNA samples. Fluorescence intensity was detected using a laser scanner (Powerscanner, TECAN, Männedorf, Switzerland), and BlueFuse Multi software was used for data processing (BlueGnome, Cambridge, UK). The “24sure microarray product description (February 8, 2012, document version 2.3, and model number 408501-00)” describes 10 Mb effective resolution for 24sure using BlueFuse software, being this the minimum size specified by BlueGnome for segmental aneuploidies. The entire protocol can be completed in less than 24 hours and, therefore, embryo transfer and vitrification of surplus euploid embryos can be scheduled for day 5.

### 2.5. Statistics

The chi-square test and Fisher exact test were used for comparisons between study groups with respect to percentages. Welch *t*-test was used to compare noncategorical variables. Bonferroni's correction for multiple group comparisons was applied and *P* < 0.05 was considered statistically significant. The implantation rate was defined as the percentage of embryos transferred resulting in an implanted gestational sac. The pregnancy rate per transfer was calculated as the percentage of clinical pregnancies with a fetal heart beat. The miscarriage rate was defined as the percentage of clinical pregnancies that were spontaneously miscarried before week 12 of pregnancy. The delivery rate per cycle was defined as the number of cycles with a live birth.

## 3. Results and Discussion

### 3.1. General Clinical Results

A total of 1420 CCS cycles were performed. The mean female age was 39.4 (SD 3.4). A total of 7210 embryos were analyzed and, in 7118 (98.7%) of them, amplification and further analysis were successful. A high percentage of aneuploid embryos were observed for all 24 chromosomes (77.6%), with 3.9% of them showing segmental aneuploidies defined as gains or losses of chromosome fragments with size larger than 10 Mb. A chaotic pattern was observed in 15.0% of the embryos. In 783 cycles, at least one euploid embryo was available for transfer, with a pregnancy rate of 53.4% per transfer and 29.4% per cycle. The miscarriage rate was 7.4%, and the delivery rate per cycle was 27.3%. The results from our CCS program using aCGH technology support our previous experience of the benefits of using FISH for a limited number of chromosomes. In fact, the average pregnancy rate per transfer for all indications was higher than in our previously published studies using FISH for similar indications, which produced pregnancy rates ranging between 30 and 40% [42–44]. Therefore, the incorporation of aCGH in our aneuploidy screening program has resulted in a clear increase in pregnancy and implantation rates, showing that aneuploidies for any of the 24 chromosomes can appear in preimplantation embryos and therefore can impair embryo viability and implantation.

### 3.2. Clinical Results according to the Origin of Oocytes and Embryos

Table 1 summarizes our results according to the origin of oocytes and embryos. Comparisons among the four groups showed a similar mean female age. The mean number of MII oocytes was 9.0 (SD 4.9) in the group of fresh oocytes; 10.1 (SD 4.7) in the vitrified oocytes group; 12.4 (SD 6.6) in the group of day-2 vitrified oocytes, and 12.8 (SD 6.9) in the day-3 vitrified embryo group. The informative and aneuploid embryos as well as their clinical outcomes were similar among groups, showing that vitrification had no detrimental clinical impact at any stage compared to fresh cycles. Statistical differences were only observed in the mean number of embryos analyzed (which was significantly higher for vitrified oocyte and vitrified day-2 embryo groups compared to fresh and day-3 vitrified cycle groups) and in the percentage of cycles reaching the embryo transfer stage, which was lower for vitrified oocytes compared to day-2 vitrified cycle groups.

A high proportion of cycles (62%) were performed in women who were aged 40 years or more. For this reason, in women with low ovarian response, the goal of vitrification was to accumulate a sufficient number of MII oocytes or embryos to be able to achieve embryo transfer and subsequent ongoing pregnancy. Oocyte vitrification from different stimulation cycles for oocyte accumulation has been successfully applied to low-responder patients in regular IVF cycles [45] and the introduction of vitrification in IVF programs opens new possibilities for embryo selection and CCS [46, 47]. Additionally, another recent publication showed that the process of oocyte vitrification does not increase embryonic aneuploidy and does not impact implantation [48]. An alternative for achieving an optimal number of embryos for biopsy is vitrification at the cleavage stage, which has also shown optimal performance in a long retrospective study [49]. However, a recent study of aCGH cycles showed that the cohort size was not significantly associated with the euploidy rate [50]. In this present study, the group of mixed vitrified oocytes showed the highest percentage of embryos with more than one aneuploidy (36.9%), which may be due to the older age of the women included in this group. Embryo vitrification at any of the other stages before the biopsy did not have an impact on the percentage and distribution of different types of chromosomal abnormalities. Similar aneuploidy rates were obtained in fresh cycles (77.2%) compared to mixed cycles with vitrified oocytes (79.7%), day-2 (76.8%), or day-3 (77.1%) cleavage-stage embryos; no differences were observed in pregnancy, implantation, or delivery rates. These data are comparable to those recently published regarding cycles with fresh and vitrified oocytes from our ovum donation program. Similar metabolomic profiles were also observed in embryos derived from fresh and vitrified oocytes, supporting the feasibility of accumulating oocytes or embryos for a single CCS cycle analysis [51].

### 3.3. Clinical Results according to the Comprehensive Chromosome Screening Indication

Table 2 shows the results for the different infertility indications studied. The mean number of embryos analyzed was significantly higher for the MF indication compared to the other indications, with a lower number of embryos analyzed for the AMA group (mean 4.6). The percentage of aneuploid embryos was similar for all indications below 40 years of age, with a significant increase in the AMA group (85.3%) compared to all the other groups (68.2% in RM, 67.7% in RIF, 71.5% in PTP, and 65.4% in MF). These results had an impact in the percentage of cycles with at least one euploid embryo for transfer, making it significantly lower for the AMA group (40.3%) compared to all the other indications (77.3% in RM, 79.2% in RIF, 78.8% in PTP, and 83.6% in MF). However, once embryo transfer was achieved, the chances of successful pregnancy and implantation were similar for all the mentioned indications, with a range between 46.0% and 62.9% for pregnancy rates per transfer and between 40.5% and 54.2% for implantation rates. However, the pregnancy rate per CCS cycle was significantly lower for the AMA group (19.3%) compared to the remaining indications (44.3%, 45.2%, 36.4%, and 52.1% for RM, RIF, PTP, and MF, resp.) due to the previously mentioned high transfer-cancellation rate. The delivery rate per cycle was also significantly lower for the AMA group (18.1%) compared to the other indications (38.4% in RM, 43.1% in RIF, 30.3% in PTP, and 50.9% in MF).

Despite the minimal effect of maternal age on implantation after the transfer of a euploid embryo, a negative effect on delivery rates has been described by other authors. A retrospective case-controlled study including CCS cycles with aCGH for PGS in AMA, RM, and RIF patients reported lower ongoing pregnancy rates per cycle in patients 35 years or older compared to patients less than 35 years. However, even in cycles in patients 38 years or older, the implantation, clinical pregnancy, and ongoing pregnancy rates significantly increased after CCS in these groups compared to their controls [52]. Another multicenter retrospective study described an increase in the incidence of aneuploid embryos, which correlated with increased maternal age, observing similar implantation and ongoing pregnancy rates per transfer after CCS in patients up to 42 years of age, after which these rates dramatically declined [53].

**Figure 1:**
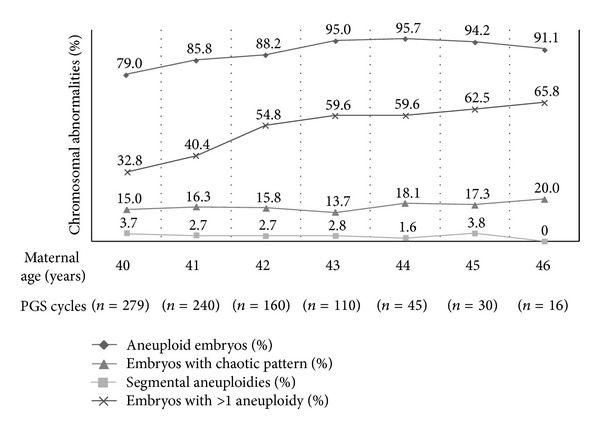
Aneuploidy rates according to maternal age in AMA group.

Interestingly, Table 2 shows a different distribution of chromosomal abnormality types according to the indication. The highest incidence of segmental aneuploidies was observed in couples with a previous chromosomally abnormal pregnancy (10.2%) and the lowest was in the AMA group (3.2%). The distribution of embryos showing a chaotic pattern was relatively homogenous among indications, with a slight decrease in RIF patients (11.2%) compared to RM (16.4%) and AMA (15.6%) patients. The most remarkable difference was observed for the percentage of embryos with aneuploidy for more than one chromosome, which was significantly higher in the AMA group (43.1%) compared to all other indications (range: 19.8%–23.5%). This percentage increases with maternal age, reaching values from 32.8% in 40 years to 65.8% in 46 years of age. Therefore, the overall incidence of aneuploidy ranges from 79.0% to 95.7% (Figure 1). This relationship between maternal age and the complexity of aneuploid errors has recently been described by Franasiak et al. (2013) [54] in a systematic report of 15169 CCS results, showing that 36% of embryos had more than one aneuploidy and that the proportion of more complex aneuploidy increases with age.

In RM couples, the transfer of euploid embryos after CCS results in a low miscarriage rate (13.3%). A multicenter study of 287 cycles in couples with idiopathic RM described 60% of the embryos as aneuploid but with a miscarriage rate of 6.9% after CCS, compared to the expected rate of 33.5% in RM control population and 23.7% in an infertile control population [28]. These results showed a clear benefit of 24-chromosome screening in couples with this etiology.

In RIF couples, previous RCTs using FISH for a limited number of chromosomes showed controversial results, with one study showing no clear benefit [55] and another showing an improvement in live birth rates compared to blastocyst transfer without previous FISH analysis [20]. Despite this, there is no RCT regarding CCS with aCGH for RIF patients, although the results described in our study support the application of aneuploidy screening for this group of patients.

In PTP couples, published data describe an increased risk of recurrent aneuploid conceptions, particularly in women under 37 years of age [56]. Previous studies on PGS with FISH analysis of a 9-chromosome panel showed high rates of abnormal embryos, ranging from 48.1% to 71.2% [57, 58], which is in agreement with the percentage of 71.5% observed in our study with 24-chromosome analysis.

Finally, we found the best clinical results after CCS in MF couples. Although, to our knowledge, there are no publications regarding CCS in MF infertile couples, this type of 24-chromosome CCS seems to be a very promising indication for this patient group, as also suggested by previous similar publications with PGS using FISH analysis [42, 59].

## 4. Conclusions

Our findings on day-3 embryo biopsies support the basis for CCS in patients in whom a high proportion of aneuploid embryos are suspected. New RCTs should be conducted in the near future to assess the feasibility of using different platforms for different clinical indications and to test for any potential increases in live birth rates resulting from more comprehensive aneuploidy screening before embryo transfer.

## Figures and Tables

**Table 1:** Clinical results of 1420 CCS cycles according to the origin of the oocytes and embryos.

	Fresh cycles	Mixed vitrified oocytes	Mixed day-2 vitrified embryos	Mixed day-3 vitrified embryos
Number of cycles	774	320	235	91
Mean female age (SD)	39.4 (3.2)	39.7 (3.9)	38.9 (3.5)	38.3 (3.4)
Mean number of embryos analyzed (SD)	4.7 (2.9)^b,c^	5.0 (2.7)^a,d,c^	6.4 (3.4)^a,b,d^	4.4 (2.7)^b,c^
Number of informative embryos	3573	1565	1499	481
Total number of aneuploid embryos (%)	2757 (77.2)	1248 (79.7)	1151 (76.8)	371 (77.1)
Number of segmental aneuploidies (%)	151 (4.2)	51 (3.2)	56 (3.7)	20 (4.1)
Number of embryos with a chaotic pattern (%)	561 (15.7)	250 (16.0)	204 (13.6)	53 (11.0)
Number of embryos with >1 aneuploidy (%)	1191 (33.3)	578 (36.9)^d^	485 (32.3)	144 (29.9)^b^
Number of embryo transfers (%)	421 (54.4)	156 (48.7)^c^	147 (62.5)^b^	59 (64.8)
Mean transferred embryos (SD)	1.4 (0.5)	1.4 (0.5)	1.5 (0.5)	1.5 (0.5)
Number of pregnancies/transfer (%)	225 (53.4)	93 (59.6)	71 (48.3)	29 (49.1)
Number of pregnancies/cycle (%)	225 (29.1)	93 (29.1)	71 (30.2)	29 (31.9)
Implantation rate	48.1	51.9	40.0	43.7
Miscarriage rate	6.6 (15/225)	8.6 (8/93)	8.4 (6/71)	6.9 (2/29)
Delivery rate/cycle	27.1 (210)	26.6 (85)	27.7 (65)	29.7 (27)

^a^ *P* < 0.05 versus fresh cycles; ^b^ *P* < 0.05 versus mixed vitrified oocytes; ^c^ *P* < 0.05 versus mixed day-2 vitrified embryos; ^d^ *P* < 0.05 versus mixed day-3 vitrified embryos. Chi-square test, Fisher exact test, or Welch *t*-test with Bonferroni's correction.

**Table 2:** Clinical results of 1420 CCS cycles according to the different indications.

	RM <40 yrs	RIF <40 yrs	PTP <40 yrs	MF <40 yrs	AMA ≥40 yrs
Number of cycles	203	188	33	116	880
Mean female age (SD)	35.9 (2.7)	36.5 (2.5)	36.8 (2.4)	34.8 (3.2)	41.5 (2.1)
Mean number of embryos analyzed (SD)	5.5 (3.1)^b,d,e^	5.9 (3.0)^a,d,e^	5.7 (3.8)^d,e^	6.8 (3.7)^a,b,c,e^	4.6 (2.6)^a,b,c,d^
Number of informative embryos	1099	1064	186	797	3972
Total number of aneuploid embryos (%)	750 (68.2)^e^	720 (67.7)^e^	133 (71.5)^e^	521 (65.4)^e^	3403 (85.3)^a,b,c,d^
Number of segmental aneuploidies (%)	61 (5.5)^e^	40 (3.7)^c^	19 (10.2)^b,d,e^	32 (4.0)^c^	126 (3.2)^a,c^
Number of embryos with a chaotic pattern (%)	180 (16.4)^b^	119 (11.2)^a,e^	24 (12.9)	125 (15.5)	620 (15.6)^b^
Number of embryos with >1 aneuploidy (%)	237 (21.6)^e^	250 (23.5)^e^	41 (22.0)^e^	158 (19.8)^e^	1711 (43.1)^a,b,c,d^
Number of embryo transfers (%)	157 (77.3)^e^	149 (79.2)^e^	26 (78.8)^e^	97 (83.6)^e^	354 (40.3)^a,b,c,d^
Mean transferred embryos (SD)	1.5 (0.5)	1.5 (0.6)	1.4 (0.5)	1.5 (0.5)	1.3 (0.5)
Number of pregnancies/transfer (%)	90 (57.3)	85 (57.0)	12 (46.0)	61 (62.9)	170 (48.0)
Number of pregnancies/cycle (%)	90 (44.3)^e^	85 (45.2)^e^	12 (36.4)	61 (52.1)^e^	170 (19.3)^a,b,d^
Implantation rate	47.9	50.9	40.5	54.2	42.4
Miscarriage rate	13.3	4.7	16.7	3.3	6.5
Delivery rate/cycle	38.4 (78)^e^	43.1 (81)^e^	30.3 (10)	50.9 (59)^e^	18.1 (159)^a,b,d^

RM: recurrent miscarriage; RIF: repetitive implantation failure; MF: severe male factor; PTP: previous trisomic pregnancy; AMA: advanced maternal age.

^a^ *P* < 0.05 versus RM <40 yrs; ^b^ *P* < 0.05 versus RIF <40 yrs; ^c^ *P* < 0.05 versus PTP <40 yrs; ^d^ *P* < 0.05 versus MF <40 yrs; ^e^ *P* < 0.05 versus AMA ≥40 yrs. Chi-square test, Fisher exact test, or Welch *t*-test with Bonferroni's correction.
